# Diagnostic value of hybrid FDG-PET/MR imaging of chronic osteomyelitis

**DOI:** 10.1186/s41824-022-00125-6

**Published:** 2022-08-01

**Authors:** Dennis Jan Willem Hulsen, Cristina Mitea, Jacobus J. Arts, Daan Loeffen, Jan Geurts

**Affiliations:** 1 Department of Orthopaedic Surgery, Research School CAPHRI, Maastricht University Medical Centre, Maastricht, The Netherlands; 2 Department of Medical Physics, Jeroen Bosch Ziekenhuis, ‘s-Hertogenbosch, The Netherlands; 3 Department of Radiology and Nuclear Medicine, Maastricht University Medical Centre, Maastricht, The Netherlands

**Keywords:** Osteomyelitis, PET/MRI, Diagnosis, Accuracy, Standardized uptake values

## Abstract

**Background:**

Magnetic resonance imaging (MRI) and 2-[^18^F]-fluoro-2-deoxy-d-glucose (^18^F-FDG) Positron Emission Tomography, paired with Computed Tomography (PET/CT) are commonly used modalities in the complicated diagnostic work-up of osteomyelitis. PET/MRI is a relatively novel hybrid modality with suggested applications in bone infection imaging, based on expert opinion and previous qualitative research. ^18^F-FDG PET/MRI has the advantages of reduced radiation dose, more soft tissue information, and is deemed more valuable for surgical planning compared to ^18^F-FDG PET/CT. The goal of this study is to quantitatively assess the diagnostic value of hybrid ^18^F-FDG PET/MRI for chronic osteomyelitis.

**Methods:**

A retrospective analysis was performed by a nuclear medicine physician and radiologist on 36 patients with ^18^F-FDG PET/MRI scans for suspected osteomyelitis. Sensitivity, specificity, and accuracy were determined with the clinical assessment by the orthopaedic surgeon (based on subsequent intraoperative microbiology or long-term follow-up) as the ground truth. Standardized uptake values (SUV) were measured and analysed by means of receiver operating characteristics (ROC).

**Results:**

This first study to quantitatively report the diagnostic value of ^18^F-FDG PET/MRI yielded a sensitivity, specificity, and accuracy of 78%, 100%, and 86% respectively. Area under the ROC curve was .736, .755, and .769 for the SUVmax, target to background ratio, and SUVmax_ratio respectively. These results are in the same range and not statistically different compared to diagnostic value for ^18^F-FDG PET/CT imaging of osteomyelitis in literature.

**Conclusions:**

Based on the aforementioned advantages of ^18^F-FDG PET/MRI and the diagnostic value reported here, the authors propose ^18^F-FDG PET/MRI as an alternative to ^18^F-FDG PET/CT in osteomyelitis diagnosis, if available.

## Background

Infection of bone and/or bone marrow, termed osteomyelitis, can cause a long-lasting burden for patients that suffer from it. It is characterized by inflammatory destruction and apposition of bone, leading to deformity and function loss. Osteomyelitis can be caused by trauma, a contiguous source of infection, or haematogenous spread of pathogens. Gram negative anaerobic bacteria are the most common pathogen that cause the infection, and the lower extremities are the most regularly affected body parts (Sheehy et al. 2010; Kaim et al. 2002). Varying causative microorganisms, anatomic area, route of contamination, duration of the infection, and patient characteristics result in highly heterogeneous clinical manifestations of osteomyelitis. Timely treatment is essential to prevent progressive bone necrosis and bone function loss, and to plan an adequate therapeutic strategy the treating physician has to make an accurate diagnosis. Acute osteomyelitis can often be treated with systemic antibiotic administration, but could progress into chronic osteomyelitis if treatment fails. Chronic osteomyelitis frequently requires surgical debridement combined with local and systemic antibiotics (Parsons and Strauss 2004). The gold standard diagnostic modality is intraoperative microbiology, requiring a surgical intervention (Lew and Waldvogel 2004). Serum markers of inflammation are used routinely in the diagnostic process, but these lack sensitivity in low-grade chronic infections and are more suitable for trend observation (Harris et al. 2011; Husain and Kim 2002).

For a preliminary clinical diagnosis and to effectively prepare surgical treatment, medical imaging plays an important role. Various imaging modalities can be used in the evaluation of suspected (chronic) osteomyelitis.

Conventional X-ray imaging is generally part of the standard work-up. However, it is nonspecific and it is only able to depict latent effects of osteomyelitis on bone anatomy. Computed tomography (CT) has excellent resolution, but its poor soft-tissue contrast makes that it provides limited insight in the active infection, depicting only latent, osseous effects similarly to conventional X-ray. Magnetic resonance imaging (MRI) is the modality of choice to image the soft tissues (muscle, marrow, and edema), that are key to diagnosing osteomyelitis (Lee et al. 2016). While X-ray, CT, and MRI depict anatomical signs of osteomyelitis, nuclear medicine techniques can image specific physiological mechanisms. Different nuclear medicine techniques are usable in osteomyelitis diagnosis. Of the various techniques 2-[^18^F]-fluoro-2-deoxy-d-glucose (^18^F-FDG) positron emission tomography (PET) generally has a high sensitivity and specificity reported in literature, and it is relatively widely available (Wang et al. 2011; Wenter et al. 2016). In 2019 a consensus document from various European Scientific Societies was published on the diagnosis of peripheral bone infection in adults, in which a prominent role was posed for both MRI and ^18^F-FDG PET (Glaudemans et al. 2019; Sconfienza et al. 2019).

Hybrid imaging development combining a nuclear imaging modality with CT has significantly advanced nuclear medicine in general, and nuclear imaging of osteomyelitis in particular (Bruggen et al. 2010). CT added anatomical reference, novel attenuation correction methods, and to some extent diagnostic information to nuclear imaging. Recently, hybrid PET/MRI scanners have become available, combining metabolic imaging on PET with the high-resolution soft tissue imaging from MRI. With the superior diagnostic value of MRI versus CT in orthopaedic infections, hybrid PET/MRI has been recognized in literature to potentially become an important imaging technique in this field (Subramaniam et al. 2017; Kouijzer et al. 2018; Fahnert et al. 2016). In 2019, we published a case series of five patients that underwent a single-injection/dual-acquisition protocol with ^18^F-FDG PET/CT and ^18^F-FDG PET/MR with the goal to qualitatively compare the two modalities in both diagnosis and operative planning of chronic peripheral osteomyelitis (Hulsen et al. 2019). We concluded that PET/MRI provided at least the same diagnostic information as PET/CT, and that PET/MRI is a more valuable modality for surgical planning. On PET/MRI, the location of infection based on ^18^F-FDG uptake could clearly be correlated with certain soft tissue structures (oedema, fluid collection, or muscle), which is paramount for surgical planning. Additionally, PET/MRI leads to a reduced radiation dose and more soft tissue information in general. Following these promising results, PET/MRI has routinely been implemented in the diagnostic work-up of osteomyelitis.

The goal of this study is to quantitatively evaluate the performance of ^18^F-FDG PET/MRI for the diagnosis of chronic osteomyelitis.

## Methods

### Patient population

We retrospectively identified all patients that were referred to PET/MRI for evaluation of suspected chronic osteomyelitis between 01-02-2015 and 01-02-2020 at the Maastricht University Medical Centre. The specific diagnosis for these patients with conventional imaging and/or clinical examination had remained inconclusive, but there was a high suspicion for chronic osteomyelitis. For all patients, surgical intervention was indicated unless PET/MRI would provide a negative result. Patients were excluded if they were under 18 years old, demented, pregnant, nursing or had recent previous surgery, fracture, or implants in the region of interest. Patients with scans that lacked MRI series according to our institute’s current standard were excluded. If patients had multiple eligible PET/MRI scans in this time period, only the first scan was included. This study is an extension of the previously reported case series, with approval of the local medical-ethical review committee (reference METC 16-4-150.1/ab, Maastricht University Medical Centre, Maastricht, Netherlands). Our institute is a designated referral centre for osteomyelitis treatment.

### Image acquisition

The patients were administered a weight-adjusted dose of 2 MBq/kg ^18^F-FDG intravenously. The radiopharmacon was acquired from a commercial radiopharmacy (GE Healthcare Radiopharmacy, Eindhoven, Netherlands). Patient blood glucose levels were confirmed to be < 10 mmol/l prior to scanning. Acquisition of the PET/MRI scan commenced 60 min after ^18^F-FDG administration. The scans were acquired with a Siemens Healthcare Biograph mMR system. The scanner has an axial field of view (FOV) of 25.8 cm, 65.6-cm ring diameter, a NEMA-specified spatial resolution near FOV centre of 4.4 mm, and sensitivity near FOV centre 13,200 cps/MBq. PET scans were acquired for a single bed position, with duration extended to the duration of MRI acquisition, ranging from 10 to 20 min. Attenuation maps were obtained by a four-tissue (air, soft tissue, fat, and lung) Dixon-volume-interpolated mode (Martinez-Möller et al. 2009). All attenuation maps were qualitatively examined visually during the scanning process (Ladefoged et al. 2014). Acquired images were corrected for scatter, attenuation, and point spread function, and reconstructed in a 344 × 344 matrix with OSEM iterative reconstruction, three iterations and 21 subsets with a 4-mm Gauss filter.

Standard diagnostic MRI sequences were acquired: T1 weighted turbo spin echo (TSE), Gadolinium contrast-enhanced fat saturated T1, T2 weighted TSE with fat saturation, Proton Density weighted images with fat saturation, and Short T1 Inversion Recovery (STIR), with additional series and acquisition directions based on anatomical location and protocol optimization over time (Kapoor et al. 2007). A flexible MRI receiver coil was used that is specifically designed for use in PET/MRI.

### Image assessment

All PET scans were assessed by an experienced nuclear medicine physician (CM), and MRI scans were assessed by an experienced radiologist (DL). Both specialists were specifically trained for musculoskeletal imaging. The assessment was reported dichotomous: either negative or positive for osteomyelitis. PET criteria used for the diagnosis of osteomyelitis were focally increased ^18^F-FDG uptake within bone or bone marrow, with higher uptake than in surrounding soft tissue. MRI was used by the nuclear medicine physician for anatomical reference, and not for diagnosis.

MRI criteria used for the diagnosis of osteomyelitis included: focally decreased bone marrow signal intensity on T1 weighted images, focally increased signal intensity of bone or bone marrow on fat-suppressed T2 weighted images, or focal enhancement of bone or bone marrow on contrast-enhanced images. Presence of secondary signs of infection such as a cortical disruption, sequester, or fistula increased the suggestion of osteomyelitis. Findings that were considered to exclude the diagnosis of osteomyelitis included normal bone marrow signal intensity on both T1- weighted and fat-suppressed T2 weighted images.

If the PET and MRI conclusions did not agree, the imaging specialists performed a joint reading to reach a consensus conclusion. During the image assessment process, the imaging specialists were blinded for clinical and laboratory patient data.

### Standardized uptake values

The following ^18^F-FDG Standardized Uptake Values (SUV) were measured for all scans:

- The local maximum SUV in the bony lesion, or expected location of the lesion (SUVmax)
- The local maximum SUV of an internal reference region (SUVmax_reference_)
- The mean SUV in an internal reference region (SUVmean_reference_)

The contralateral noninfected site was used as internal reference region when possible. If no images of the non-infected side were available, a the reference region was used in the same side as the infection with a visually normal appearance on PET and MRI. The measurements were performed in manually placed regions of interest with the region of interest of the internal reference region delineated as big as the area with homogenous uptake allowed to limit statistical variation.

Additionally, SUV ratios were calculated:

- SUVmax ratio for bone: SUVmax/SUVmax__reference_
- Target to background ratio (TBR): SUVmax/SUV_mean_reference_

### Clinical assessment

The patients were all assessed clinically by an experienced orthopaedic surgeon with experience in treatment of orthopaedic infections (JG). The surgeon assessed both the presence of infection (including soft tissue infections) and the presence of osteomyelitis specifically. Osteomyelitis was diagnosed based on positive intraoperative bone microbiology assessment or uneventful clinical and radiological follow-up of at least one year. For those patients that underwent surgery, the surgery (during which bone microbiology was acquired) typically occurred 6–8 weeks after the corresponding PET/MRI scan. In accordance with international guidelines (Oxford protocol: separate instruments for each sample, no‐touch technique, no suction until samples are taken), three intraoperative cultures were taken from the bone, and 2 from soft tissue (Bose et al. 2015; Mcnally and Sendi 2015). A distinction was made between causative organisms with a highly virulent or low virulent nature (Beam and Osmon 2018). In case of a diagnosis based on clinical follow-up, the distinction between osteomyelitis and soft tissue infection was based on deep bone pain, pain on percussion, and pain on load bearing.

### Statistical assessment

PET/MRI conclusions for osteomyelitis were validated as either true positive, false positive, true negative, or false negative with the clinical assessment as the ground truth. These results were quantitatively analysed to produces sensitivity, specificity, positive predictive value (PPV), negative predictive value (NPV), and accuracy for the two modalities separately, and for PET/MRI combined. For these results, a 95% Clopper–Pearson confidence interval was calculated.

For analysis of the SUV measurements, a standard t-test was used to compare means between the infected and noninfected and between the osteomyelitis and no osteomyelitis group. Receiver Operating Characteristic (ROC) curves for SUV parameters were calculated and the most suitable cut-off value for positivity was determined by selecting the value geometrically closest to a sensitivity and specificity of 100%. SPSS Statistics 27 (IBMCorp., Armonk NY) was used for all statistical assessments.

## Results

### Patient population

In the selected timeframe, 36 eligible cases were found (Table 1). The average age of the patients was 56 years. 15 patients were female (42%) and 21 were male (58%). In 29 cases (81%) the suspected infection was located in the lower extremities: 9 metatarsal, 8 tibia, 7 femur, 2 calcaneus, 2 cuboid, 1 ankle. 5 cases (14%) were located in the upper extremities: 3 finger, 1 humerus, 1 ulna. The remaining 2 were located in the pelvis and sacrum.

**Table 1:** Patient characteristics

	No infection (*N* = 6)	Soft tissue infection (*N* = 7)	Osteomyelitis (*N* = 23)	Total cases (*N* = 36)
Diagnosis confirmed by				
Follow-up only	6	6	12	24
Microbiology only	0	0	0	0
Both	0	1	11	12
Gender				
Female	4	2	9	15
Male	2	5	14	21
Mean age (years)	54	60	56	56
Location of infection				
Metatarsal	1	2	6	9
Tibia	2	1	5	8
Femur			7	7
Finger	1		2	3
Calcaneus		2		2
Cuboid		1	1	2
Pelvis	1			1
Ankle			1	1
Sacrum	1			1
Humerus		1		1
Ulna			1	1
Source of infection				
Bone injury	2	2	12	16
Contiguous source	2	5	8	15
Haematogenous	2		3	5
Causative agent virulence				
Low		1	5	6
High			7	7
Mean time since supposed causative event (years)	6	3	12	9

The columns show the cases broken down into the three categories of final clinical diagnosis In one case, the causative agent was known from open wound culture during clinical follow-up

The final diagnosis by the orthopaedic surgeon based on follow-up or intraoperative microbiology was osteomyelitis in 23 cases, soft tissue infection in 7 cases, and no infection in 6 cases. In 13 cases intraoperative microbiology was acquired, while the diagnosis of the 23 other cases relied only on follow-up. Of the 23 cases with final diagnosis osteomyelitis, 12 were based on follow-up and 11 on both follow-up and microbiology. One case was labelled a soft tissue infection based on microbiology, while the other 6 soft-tissue infection cases were only based on follow-up. In one case, the intraoperative microbiology remained negative while the perioperative image was very suggestive of osteomyelitis. Based on the operative experience and follow-up of the surgeon, this case was concluded to be culture-negative osteomyelitis.

Bone injury was the most common cause of infection with 16 cases, a contiguous source of infection was the cause in 15 cases, and haematogenous spread in 5 cases. The time between the causative event and PET/MR scan was 9 years on average, significantly higher in the osteomyelitis group (11.8 years) compared to the patients with no osteomyelitis (4.6 years, *p* = 0.015).

### Image assessments

**Fig. 1:**
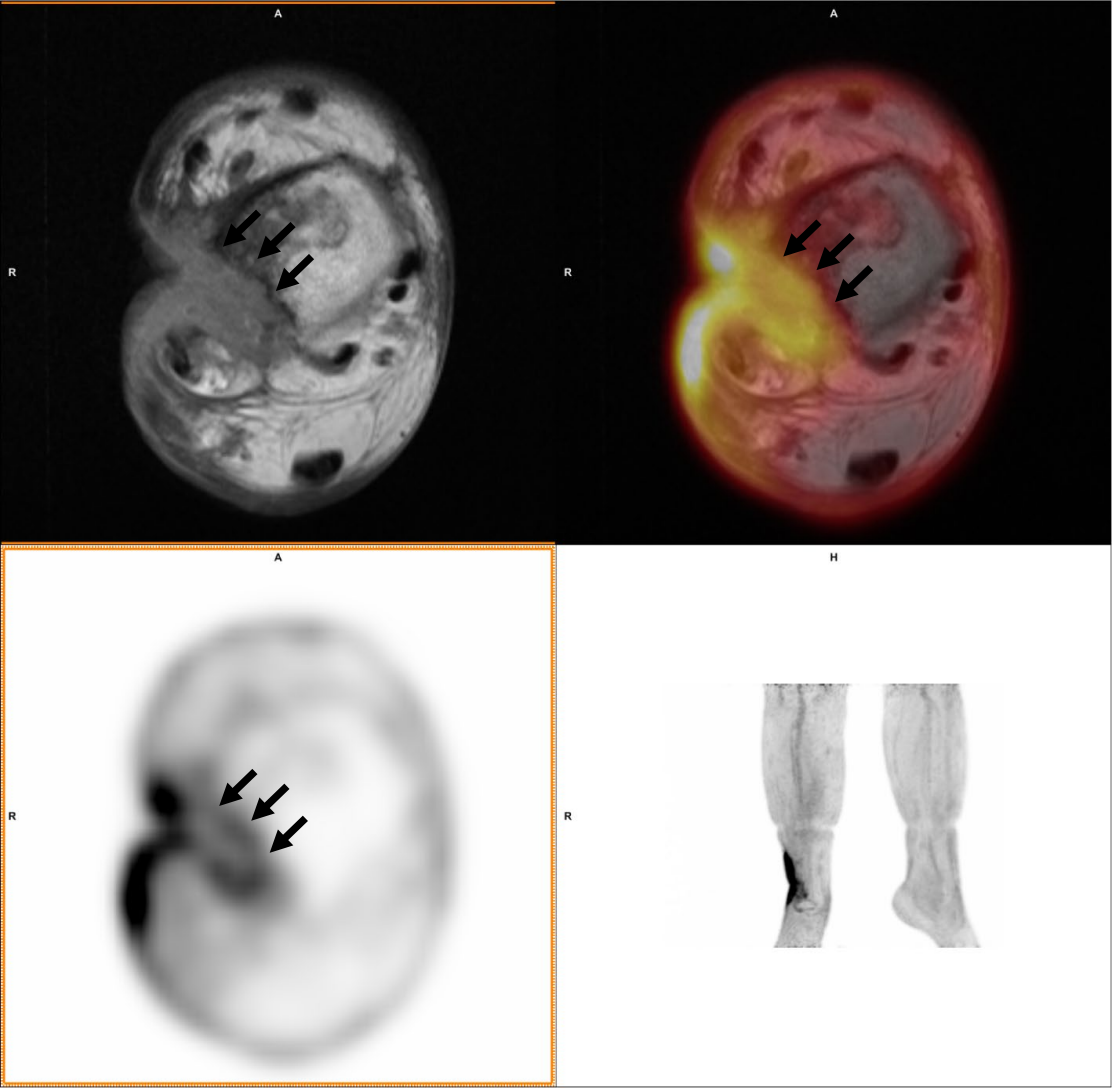
A 65 year old woman with a 4 year old bone injury. Based on MRI, induration of soft tissue was found and the radiologist assessed that the ossal defect was pre-existing with only soft tissue remaining in the defect, leading to the conclusion of soft tissue infection. PET did reveal increased FDG uptake down to the bone (arrows). In the consensus reading this was concluded to be osteomyelitis. The final clinical diagnosis was (culture-negative) osteomyelitis based on the perioperative experience and over 3 years follow-up

**Fig. 2:**
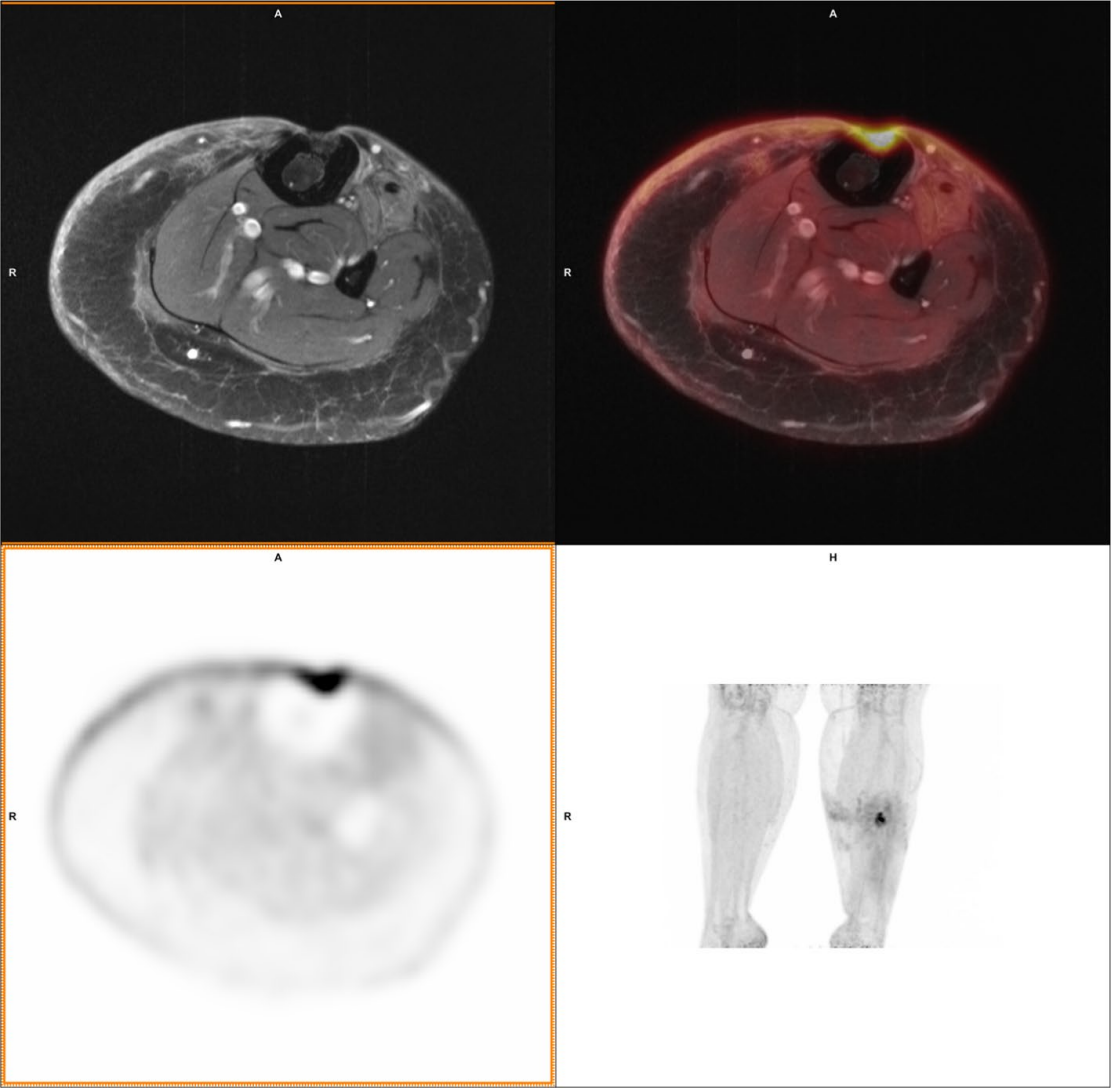
A 33 year old woman with a 2 year old soft tissue trauma as a suspected contiguous source of osteomyelitis in the tibia. FDG accumulation was measured in the defect, and the cortex was assessed to be involved based on the PET scan. A lack of signs for oedema and (aside from the pre-existing defect) a seemingly intact cortex on MRI resulted in a consensus PET/MRI diagnosis of soft tissue infection. This was contradicted by the final clinical diagnosis (osteomyelitis) based on microbiology and clinical follow-up

^18^F-FDG PET/MRI yielded 13 True Negative (TN), 18 True Positive (TP), 5 False Negative (FN), and 0 False Positive (FP) results, resulting in a sensitivity, specificity, accuracy, NPV, and PPV of 78%, 100%, 86%, 72%, and 100% respectively. In 30 cases, the PET and MRI conclusions agreed directly. In the other 6 cases, a consensus reading led to the final PET/MRI conclusion. This conclusion was TP in 4 cases, and FN in 2 cases. Of the latter 2, MRI had the correct initial conclusion in one case, and PET in the other. Figures 1 and 2 present examples of a case in which a consensus reading was required to take the final conclusion.

### Standardized uptake values of osteomyelitis

**Fig. 3:**
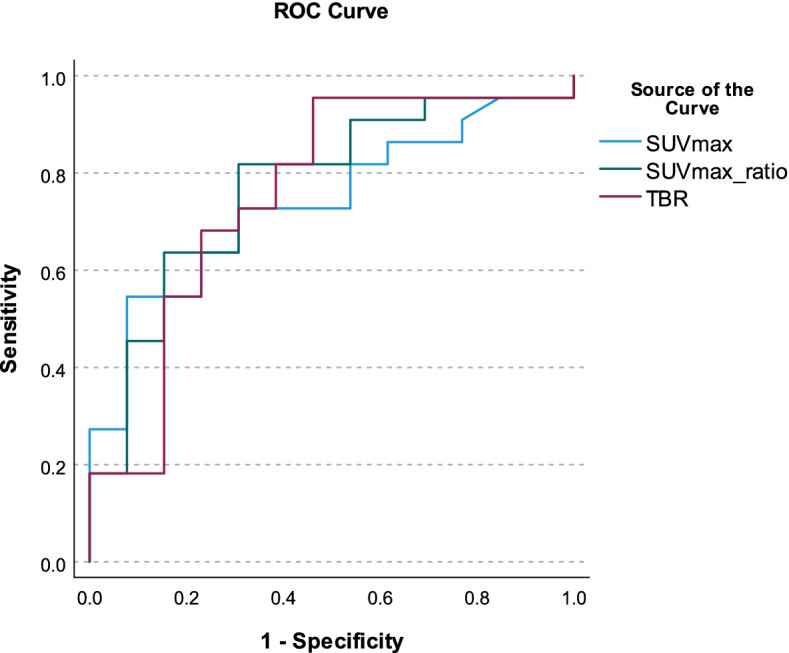
ROC curves for the SUV parameters using the presence of osteomyelitis determined by the gold standard as the state variable

Results for analysis of the SUV parameters comparing the groups with and without osteomyelitis as determined by the clinical assessment are shown in Table 2 and Fig. 3. Mean SUVmax was 6.8 in the osteomyelitis and 2.0 in the no osteomyelitis group (*p* = 0.067), with an area under the curve (AUC) of 0.736 and an optimal threshold of 2.0 yielding 72.7% sensitivity and 69.2% specificity. Mean SUVmax_ratio was 20.6 in the osteomyelitis and 8.6 in the no osteomyelitis group (*p* < 0.05), with an AUC of 0.769 and an optimal threshold of 7.5 yielding 81.8% sensitivity and 69.2% specificity. Mean TBR was 37.1 in the osteomyelitis and 14.4 in the no osteomyelitis group (*p* < 0.05), with an AUC of 0.755 and an optimal threshold of 18.9 yielding 68.2% sensitivity and 76.9% specificity. SUVmax, TBR, and SUVmax_ratio were all higher in the affected group compared to the unaffected group.

**Table 2:** Results for the analysis of SUV parameters using the presence of osteomyelitis determined by the gold standard as the state variable

	Mean				ROC analysis		
	Osteomyelitis (*N* = 23)	No osteomyelitis (*N* = 13)	*p* value of T-test	AUC of ROC	Best cut-off value	Sensitivity (%)	Specificity (%)
SUVmax	6.8	2.0	.067	.736	2.0	72.7	69.2
SUVmax_ratio	20.6	8.6	.049*	.769	7.5	81.8	69.2
TBR	37.1	14.4	.026*	.755	18.9	68.2	76.9

Mean SUV values were higher for the cases with high virulent microorganisms (SUVmax 1.3, SUVmax_ratio 6.1, TBR 10.4) than for the low virulent cases (SUVmax 5.4, SUVmax_ratio 23.0, TBR 51.6). TBR showed a significant difference with a p value for the T-test < 0.05. SUVmax and SUVmax_ratio comparison between the two groups neared significance, with *p* = 0.091 and *p* = 0.054 respectively (Table 3).

**Table 3:** Results for the analysis of SUV parameters in cases with low and highly virulent causative agents

	Mean		
	Low virulence (*N* = 6)	High virulence (*N* = 7)	*p* value of T-test
SUVmax	1.3	5.4	.091
SUVmax_ratio	6.1	23.0	.054
TBR	10.4	51.6	.037*

## Discussion

Patients with suspected chronic osteomyelitis present a difficult task to the clinician who has to initiate adequate therapy to prevent deteriorating bone function loss and serious discomfort. The clinical diagnosis of chronic osteomyelitis is difficult to make and the gold standard diagnostic modality, deep tissue culture microbiology, requires an intervention. Imaging therefore plays an important role in the diagnostic work-up of osteomyelitis. In recent literature, ^18^F-FDG PET/MRI as a novel hybrid imaging modality has been identified to have a potential application in imaging osteomyelitis. In 2019, the current authors reported a case series to qualitatively compare ^18^F-FDG PET/MRI to the common ^18^F-FDG PET/CT in diagnosis and preoperative planning of osteomyelitis (Hulsen et al. 2019). The study presented here follows up on the case series by quantitatively evaluating the accuracy of ^18^F-FDG PET/MRI in the diagnosis of suspected osteomyelitis, and to our knowledge it is the first to do so.

The cohort in this study consisted of 36 patients with suspected osteomyelitis. PET/MRI was performed to support the clinical treatment strategy. The PET/MRI results were validated by intraoperative cultures 6–8 weeks after imaging, or if no surgery was carried out, by clinical follow-up of at least 1 year. Our department is a tertiary referral centre for osteomyelitis treatment. A substantial proportion of the patients suffer from a chronic, recurring, and/or low-grade infection, which is illustrated by the long duration of complaints in this cohort: 9 years on average. This makes the presented cohort similar to the extensive PET/CT cohort described by Wenter et al. (Wenter et al. 2016).

**Fig. 4:**
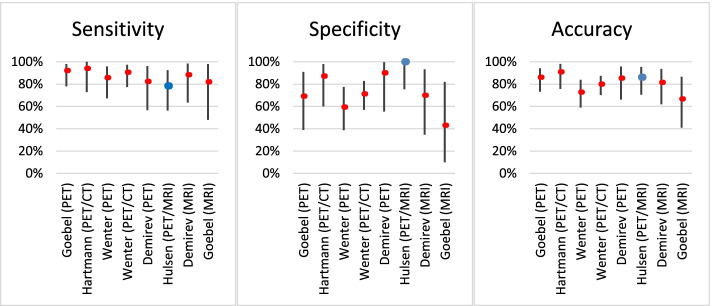
Results for Sensitivity, specificity, and accuracy for the diagnosis of osteomyelitis with 18F-FDG PET(/CT or /MRI) and MRI of the current study compared to results from literature. Bars indicate 95% CI

The availability of literature to compare our results with is limited. In the past decades, only a handful of peer-reviewed papers have emerged that provide new results regarding ^18^F-FDG PET use in osteomyelitis. This is also apparent from the meta-analyses of Termaat et al. in 2005 that included 4 studies, Wang et al. in 2011 with 7, and Govaert et al. in 2017 with 6 included (Wang et al. 2011; Govaert et al. 2017; Termaat et al. 2005). Of the combined total included studies from these three meta-analyses, some are deemed unsuitable for comparison with our results because they are dated, and scanning techniques have greatly improved since their publication. Moreover, due to the limited number of cases, some studies have a statistical uncertainty in their results that is so wide that comparison has no added value. From the studies in the aforementioned meta-analyses, three were found suitable to compare our results with (Wenter et al. 2016; Goebel et al. 2007; Hartmann et al. 2007). A previous study from our own institute has been added, whose patient cohort and approach are comparable to the current study (Demirev et al. 2014). Table 4 and Fig. 4 show the sensitivity, specificity, and accuracy of the selected studies next to our results, with a 95% Clopper-Pearson Confidence Interval (CI) to indicate statistical uncertainty.

**Table 4:** Results for Sensitivity, specificity, and accuracy for the diagnosis of osteomyelitis with ^18^F-FDG PET(/CT or /MRI) and MRI of the current study compared to results from literature

	Modality	Cases	Year	Sensitivity (95% CI)	Specificity (95% CI)	Accuracy (95% CI)
Goebel	^18^F-FDG PET	50	2007	92% (78–98%)	69% (39–91%)	86% (73–94%)
Hartmann	^18^F-FDG PET/CT	33	2006	94% (73–100%)	87% (60–98%)	91% (76–98%)
Wenter	^18^F-FDG PET	55	2016	86% (67–96%)	59% (39–78%)	73% (59–84%)
Wenter	^18^F-FDG PET/CT	94	2016	90% (77–97%)	71% (57–83%)	80% (70–87%)
Demirev	^18^F-FDG PET	27	2014	82% (57–96%)	90% (56–100%)	85% (66–96%)
Hulsen	^18^F-FDG PET/MRI	36	2021	78% (56–93%)	100% (75–100%)	86% (71–95%)
Demirev	MRI	27	2014	88% (64–99%)	70% (35–93%)	81% (62–94%)
Goebel	MRI	18	2007	82% (48–98%)	43% (10–82%)	67% (41–87%)

From the study by Wenter et al., only the results for cases without an implant were used for valid comparison

The sensitivity, specificity, and accuracy of our study are comparable to the results by the other studies with the 95% CI showing little statistical differences. Our sensitivity of 78% is slightly lower than the range of the other studies (range 82–94%). Two important possible causes for this relatively low sensitivity can be identified. First of all, this finding could be attributed to the highly selected patient cohort with low grade causative agents. Lankinen et al. found that in leporine experimental osteomyelitis SUV values for *Staphylococcus aureus* lesions, a high grade causative agent, were double to that from Staphylococcus Epidermis lesions, a low grade agent (Lankinen et al. 2012). Of the 13 culture-confirmed cases in our study, 7 were caused by highly virulent pathogens, and 6 by low grade pathogens. Among the five false negative PET/MRI cases, three were caused by low virulent pathogens. Second, our image assessments have been performed by only one radiologist and one nuclear medicine physician to simulate regular clinical assessment. Other studies have used a combined assessment by multiple specialists, which could have increased the sensitivity.

The specificity of 100% that we reported is remarkably higher than the specificity of the other studies, which had a range of 51–90% for ^18^F-FDG PET(/CT). It has been well described in literature that ^18^F-FDG-PET is very nonspecific for many pathological processes as malignancies, inflammation, and infection all have a high ^18^F-FDG uptake (Glaudemans et al. 2015). With the addition of diagnostic MRI, a lot more context is gained on the soft tissues around a region with high ^18^F-FDG uptake, which could help to make a more specific diagnosis. Moreover, advancements in PET resolution and high contrast anatomical reference make that the location of the lesion can be identified more accurately, in order to discriminate between osteomyelitis and a soft tissue problem. In our cohort, not a single case of the seven soft tissue infections was wrongly identified as osteomyelitis. Of the 5 FN cases, 4 patients did undergo surgery because of strong clinical suspicion of osteomyelitis despite the PET/MRI being negative for bone infection. Most of these cases did show signs of infection on PET/MRI, but the bone was not assessed to be involved.

Noteworthy is the fact that in all studies performed by other institutes, the reported sensitivity is higher than the specificity while it is the other way around in our results. In any diagnostic modality there is a trade-off between sensitivity and specificity. In the end it is the radiologist or nuclear medicine physician who determines if he or she errs on the side of caution: a high sensitivity at the cost of specificity, or the highest amount of correct readings: highest accuracy. We would argue that the accuracy of an imaging modality might be the most important parameter for the clinician planning the treatment strategy of chronic osteomyelitis because of its chronic nature. Our accuracy of 86% is among the highest of the reported studies (range 67–91%).

### SUV analysis

Quantitative image analysis can guide nuclear medicine physicians to a quicker and more reproducible image review. SUV is the main parameter for quantitative nuclear imaging analysis, and it is widely used in oncology. For infection assessment, its use has not been thoroughly validated, and its applications in this clinical field remain unsuccessful (Glaudemans et al. 2015; Jamar et al. 2013). With the heterogeneous cohort presented here (various grades of pathogens and infection) it was not to be expected that a dichotomous quantitative SUV analysis would outperform the manual assessment by a nuclear medicine physician. The results show that sensitivity and specificity with the optimal cut-off values lead to a lower accuracy than the qualitative assessment. Combining SUV to the qualitative assessment could however increase the accuracy, as Lemans et al. demonstrated in fracture related infections (Lemans et al. 2019). In our results, 2 cases that yielded a false negative PET/MR result would have been positive if SUVmax_ratio was used with the suggested cutoff value to detect osteomyelitis.

SUV measurements and the ROC analysis can be an indication for the objective quality of the PET/MRI acquisition protocols, when compared to SUV measurements for ^18^F-FDG PET imaging of osteomyelitis in literature (Table 5). The results for SUVmax in the presented cohort compare well with values reported in literature, which confirms the validity of our results (Wenter et al. 2016; Fahnert et al. 2016; Demirev et al. 2014; Familiari et al. 2011). The agreement between SUV measured by PET/MRI and PET/CT has been described by others (Lyons et al. 2015; Kershah et al. 2013).

**Table 5:** SUV measurement results of this study (first data column) compared to values reported in literature

	Hulsen	Wenter	Fahnert (spondylodiscitis)	Demirev	Familiari (diabetic foot)
*SUVmax*					
Osteomyelitis	6.8	6.6	5.1	–	–
Negative	2.0	3.7	2.7	–	–
Suggested Cutoff	2.0	3.9	–	3.0	2.0
AUC	.736	.717	–	-	–
*SUVmax_ratio*					
Osteomyelitis	20.6	5.2	–	-	–
Negative	8.6	2.8	–	-	–
Suggested Cutoff	7.5	3.0	2.1	2.0	–
AUC	.769	.702	0.95	-	–

Our SUVmax_ratio’s are considerably higher than the values reported by Wenter et al. and Fahnert et al. (Wenter et al. 2016; Fahnert et al. 2016). This could be caused by technological advancements. Improvement in reconstruction techniques and increased PET sensitivity lead to reduced partial volume effects and less noise, ultimately increasing the contrast-to-noise ratio with which these measurements are associated with. The AUC of our SUVmax_ratio ROC (.769) was higher than Wenter et al. reported, but considerably lower than Fahnert et al. The latter could have benefited from a very homogeneous study population, as it was focused only on spondylodiscitis.

### Limitations and recommendations

Our study is limited by its retrospective design with an associated selection bias, and the relatively low amount of cases. Furthermore, the final diagnosis was based only on clinical follow-up in 24/36 cases. The culture-negative case of osteomyelitis could be regarded as a deviation from the gold standard, but as any diagnostic test microbiologic cultures are also not 100% accurate. Culture-negative osteomyelitis is not uncommon according to literature (Floyed and Steele 2003). Lankinen et al. found no difference between the level of ^18^F-FDG PET uptake in cases with positive or negative bacterial cultures in patients with histologically proven osteomyelitis (Lankinen et al. 2017). This indicates that ^18^F-FDG PET could help to confirm the presence of osteomyelitis even in culture-negative cases.

With contemporary techniques, CT remains superior to MRI as an anatomical reference to clearly depict the boundary of bone. Suspected osteomyelitis is regularly located in anatomic locations with pre-existing osseous degradation, Figs. 1 and 2 show two cases with osseous degradation that is difficult to assess based on MRI. PET can clearly indicate the extent of the active infection, but MRI cannot provide a clear delineation of the bone. In these two shown cases, one was correctly identified as osteomyelitis while the other was false negative. In both cases, CT would have had a benefit over MRI for anatomical reference for the specific purpose to provide clear delineation of the bone.

It is essential for the assessment of the PET/MRI images that the physician who assesses the PET/MRI images has been trained in musculoskeletal MRI. If this is not the case for the nuclear medicine physician, cooperation between the nuclear medicine physician and musculoskeletal radiologist is key. Moreover, there are many developments to produce synthetic CT from MRI in other fields such as radiotherapy, which could also benefit PET/MRI for both attenuation correction and anatomical reference (Johnstone et al. 2018). Additionally, recent studies show that MRI-based synthetic CT can even be applied as a diagnostic tool in orthopaedics and orthopaedic infections (Florkow et al. 2021; Jans et al. 2021). This novel technology is expected to be a great asset to PET/MRI in osteomyelitis diagnosis.

In the MRI acquisition protocol for osteomyelitis in our institute the use of an intravenous Gadolinium-based contrast agent is standard practice, although it is debated in literature (Averill et al. 2009; Kan et al. 2010). Gadolinium contrast does not increase diagnostic sensitivity or specificity, but provides a better delineation of the extent of the infection (Glaudemans et al. 2019). ^18^F-FDG PET however is able to provide this same information, which decreases the added value of the contrast agent when MRI is combined with ^18^F-FDG PET. One possible advantage from Gadolinium-based contrast remains the ability to characterize an abscess in soft tissue (Hulsen et al. 2019; Averill et al. 2009).

## Conclusion

**Fig. 5:**
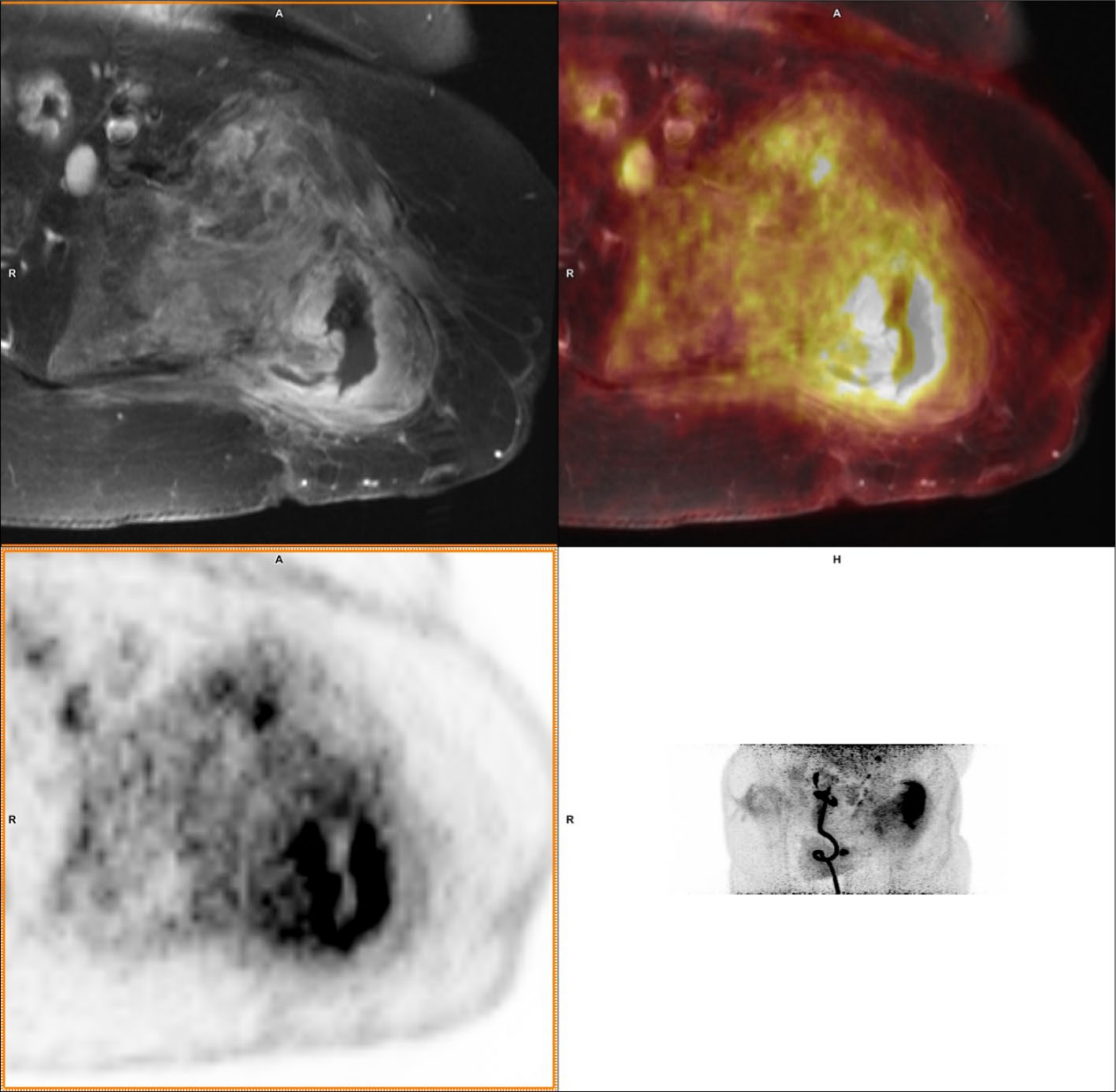
A 43 year old male with a 20 year old complex bone injury. The proximal femur was destructed and soft tissue around the bone showed high FDG uptake. PET and MRI conclusions were consistently positive for osteomyelitis. In this case the extent of the infection based on PET/MRI guides the clinician planning surgery. The presence of osteomyelitis was confirmed by both intraoperative cultures and follow-up

The presented results in this cohort of 36 patients show that the diagnostic value of ^18^F-FDG PET/MRI is comparable to that of ^18^F-FDG PET/CT in the challenging diagnosis of osteomyelitis. Additionally, the ROC showed a higher AUC for SUVmax and SUVmax_ratio than reported in literature for ^18^F-FDG PET/CT. Other advantages of PET/MRI over PET/CT include a reduced radiation dose and additional soft tissue information for surgical treatment planning (Fig. 5). The authors therefore advice physicians with a PET/MRI scanner available to consider it as an alternative for ^18^F-FDG PET/CT in osteomyelitis diagnosis, while continuously evaluating its value.

## Data Availability

The anonymized datasets generated and analysed during the current study are available from the corresponding author on reasonable request.

## Abbreviations

AUC: Area under the curve
CT: Computed tomography
FDG: 2-[^18^F]-fluoro-2-deoxy-d-glucose
FN: False negative
FOV: Field of view
FP: False positive
METC: Medical-ethical review committee
MR: Magnetic resonance
MRI: Magnetic resonance imaging
NEMA: The Association of Electrical Equipment and Medical Imaging Manufacturers
NPV: Negative predictive value
OSEM: Ordered subset expectation maximization
PET: Positron emission tomography
PPV: Positive predictive value
ROC: Receiver operating characteristic
STIR: Short T1 inversion recovery
SUV: Standardized uptake values
TBR: Target to background ratio
TN: True negative
TP: True positive
TSE: Turbo spin echo
